# Prospective study of recovery from copperhead snake envenomation: an observational study

**DOI:** 10.1186/s12873-015-0033-6

**Published:** 2015-05-15

**Authors:** Eric J Lavonas, Charles J Gerardo, for the Copperhead Snakebite Recovery Outcome Group

**Affiliations:** Rocky Mountain Poison and Drug Center, Denver Health and Hospital Authority, 777 Bannock Street, MC 0180, Denver, CO 80204 USA; Department of Emergency Medicine, University of Colorado School of Medicine, Aurora, CO USA; Division of Emergency Medicine, Duke University School of Medicine, Durham, NC USA

**Keywords:** Agkistrodon, Antivenins, Crotalid venoms, Disability evaluation, Lower extremity, Quality of Life, Recovery of function, Snake bites, Upper extremity

## Abstract

**Background:**

Although much is known about signs, symptoms, and management in the acute phase of crotaline snake envenomation, little is known about signs, symptoms, function, and quality of life during the recovery phase. The purpose of this observational pilot investigation is to evaluate the utility of several clinical outcome instruments in the setting of copperhead snakebite, and to characterize the clinical course of recovery.

**Methods:**

This is a multi-center prospective, open-label, observational study of patients envenomated by copperhead snakes. We administered the Disabilities of the Arm, Shoulder, and Hand (DASH), Lower Extremity Functional Scale (LEFS), Patient-Specific Functional Scale (PSFS), Work Productivity and Ability Impairment: Special Health Problem (WPAI: SHP), Patients’ Global Impression of Change (PGIC), Patient’s Global Assessment of Recovery (PGAR), and SF-36 instruments, obtained numeric pain rating scales, and measured grip strength, walking speed, and swelling prior to hospital discharge and 3, 7, 14, 21, and 28 days after envenomation.

**Results:**

20 subjects were enrolled; none were lost to follow-up. Most (80%) had moderate severity swelling, and most (75%) received antivenom. Across the broad range of measures, abnormalities of pain, swelling, impairments of physical and role function, and quality of life persisted for 7–14 days in most subjects. Validated self-reported outcome measures, such as the DASH, LEFS, PSFS, PGIC, SF-36, and the daily activities impairment portion of the WPAI: SHP were more responsive than measurements of swelling or walking speed. Data quality issues limited the utility of the work impairment portion of the WPAI: SHP. Residual signs, symptoms, and impairment in some subjects lasted through the 28-day study period. The study design precluded any assessment of the effectiveness of antivenom.

**Conclusions:**

Signs, symptoms, impaired function, and decreased quality of life typically last 7 – 14 days after copperhead envenomation. Several tools appear responsive and useful in studying recovery from pit viper envenomation.

**Trial registration:**

ClinicalTrials.gov NCT01651299

## Background

Approximately 8,500 people are treated in emergency departments (EDs) in the United States (US) each year for evaluation of potentially venomous snakebite [1]. The vast majority of venomous bites are caused by crotaline snakes (pit vipers: rattlesnakes, cottonmouths, and copperheads). Among envenomation cases for which the species is known, approximately 51% are due to copperheads (*Agkistrodon contortrix*) [2]. However, only rattlesnake and cottonmouth victims were enrolled in the premarketing trials for the current US pit viper antivenom, Crotalidae Polyvalent Immune Fab (Ovine) (CroFab^®^, BTG International, West Conshohocken, PA; hereafter, FabAV) [3,4]. Data from the US National Poison Data System suggest that antivenom use is much less common in copperhead victims (36% of patients treated in 2007 received antivenom) than in rattlesnake victims (55%) [5]. This is likely because copperhead envenomation patients rarely develop life-threatening systemic effects, such as hypotension and hematologic abnormalities that are common in rattlesnake envenomation [6-8]. The proportion of copperhead victims receiving antivenom has increased significantly, from 21% in 2001 to 36% in 2007, with significant regional variations [5].

Even though copperhead envenomation is rarely fatal, virtually all patients experience pain and swelling of the envenomated limb [6,8-11]. Most patients recover and resume activities of daily living within 2–4 weeks, but in a minority of cases, residual symptoms last a year or more [10-12]. Persistent limb dysfunction has been described in patients managed with and without antivenom [10-12].

Although antivenom administration clearly halts the progression of local tissue injury from snake envenomation, it is unclear whether antivenom reduces the duration or severity of lasting limb impairment [6-8,10]. The only previous investigation was a small clinical trial that was terminated early and was underpowered to detect any differences between patients receiving antivenom or placebo [10]. This question is important because FabAV is very expensive; an initial dose of 4 to 6 vials can cost a hospital approximately $US 12,000 – 21,000.

While several assessment tools exist to measure limb function, impairment, and quality of life following limb trauma and limb surgery, none of these tools have been validated in the setting of snakebites. The ability to perform valid and reliable serial assessments is a prerequisite to a clinical trial evaluating the effect of antivenom administration on recovery from copperhead snakebite.

The purpose of this pilot investigation is to evaluate the utility of several clinical outcome instruments in the setting of copperhead snakebites. A secondary objective is to characterize the clinical course of signs, symptoms, and impairment during the recovery phase of copperhead snakebite managed with and without FabAV.

## Methods

### Study design and setting

This is a multi-center, prospective observational study conducted at 11 clinical sites across the southeastern US. All treatment provided, including the decision to administer or not administer antivenom, was at the discretion of the treating physician. Subjects were enrolled in the emergency department, followed through their initial hospital encounter, and returned for outpatient assessments performed 3, 7, 14, 21, and 28 days after envenomation.

### Participants

Patients were eligible for inclusion if they were adults (aged 18 years or older) who were envenomated by a copperhead on an extremity, distal to the elbow or knee. Snake species identification was established by one of four means: snake or photograph of the snake brought to the ED, envenomation that occurred in an area where copperheads are the only endemic pit viper, envenomation by a captive copperhead, or the patient correctly identifying a copperhead from an array of snake photographs. Although clinical evidence of venom effect (limb swelling and/or tenderness) was required, the venom effects did not need to be progressing at the time of enrollment. Patients must have presented for their initial episode of care within 24 hours of envenomation, and enrollment had to be completed within 48 hours of envenomation and prior to discharge from the ED or hospital. Prisoners and women who were pregnant or breastfeeding were excluded. In addition, patients were excluded if they were unable to read and comprehend the consent document or written assessment tools or if they had a distracting injury or other condition that would limit the ability to make a reliable self-report of functionality status based solely on the condition of interest. Patients who sustained a previous snake envenomation to any body area or a previous injury to the envenomated limb within 30 days prior to enrollment were also excluded. Study materials were prepared in English and Spanish, with back-translation used to verify accuracy of Spanish-language assessment materials.

### Data collection

Following screening, enrollment, and exclusion of pregnancy, each subject provided demographic information, past medical and medication histories, history of envenomation, information about usual occupation and left-hand or right-hand dominance. A complete physical examination was also performed. Data about the initial hospital encounter, including date and time of arrival, maximal extent of swelling, antivenom administration, laboratory test results, and adverse events were recorded by study personnel at the time of care, and missing information was obtained from the hospital record.

Formal study assessments were performed prior to discharge from the initial ED/hospital encounter and then 3 (+/−1) days and 7, 14, 21, and 28 (+/− 3) days after envenomation. At each study assessment, subjects were asked to provide a detailed history of all medications taken since the previous study visit, and a focused physical assessment was performed to evaluate the extent of swelling and the presence or absence of necrotic tissue. The results of any laboratory studies were captured, and the subject was questioned about potential adverse events from treatment or study participation. Following this data collection, study-specific assessments were performed, as detailed below. *A priori* procedures were established to deal with subjects who were unable to attend scheduled follow-up visits as detailed below.

### Assessment instruments

Study-specific assessments were performed in the following order:

#### Analgesic use

Using data from the a standardized list of medications supplemented by focused questions as needed, study personnel categorized the subject’s analgesic use in the previous 24 hours as: no analgesic; non-prescription analgesics only; prescription, non-opioid analgesics; opioid analgesics (including tramadol and opioid combination products). The results were analyzed as ordinal data.

#### Recovery: patient global assessment of recovery

This is a single item question that is administered verbally. The question is, “Have you completely recovered from your snakebite?” The purpose of this instrument was to test whether response to this single question was a useful proxy for more involved assessments of recovery.

#### Pain: numeric pain rating scale

This is a single item question administered verbally. The question is, “Please rate your pain on a scale of 0 to 10, with 0 being no pain and 10 being worst possible pain.” Ordinal (Likert) scales are well-correlated with visual analog scale scores for muscle soreness and are more easily administered [13].

#### Swelling: numeric swelling scale

This is a single item question administered verbally. The question is, “Please rate your swelling on a scale of 0 to 10, with 0 being no swelling and 10 being very severe swelling.” The purpose of this instrument was to test whether response to this single question was a useful proxy for objective assessments of swelling.

#### Resumption of work, school, or usual activities of daily living (ADLs)

This is a two-stage question that is administered verbally. The question is, “Have you returned to full duties or participation at [state subject’s occupation]?” If the subject answered in the affirmative, then the answer to a follow-up question, “What was the first day that you returned to full duties or participation at [occupation]?” was recorded. The purpose of this instrument was to test whether response to this single instrument was a useful proxy for more involved assessments of ability to perform work or ADLs. This instrument was first administered on the Day 3 assessment.

#### Recovery: Patient’s Global Impression of Change (PGIC)

This is a two-item assessment tool that uses separate ordinal scales to assess change “since beginning treatment at this clinic” [14]. The first item, a 7-item Likert scale, anchored at 1 (“No change (or condition has gotten worse)”), is used to assess improvement. The second item is an 11-item Likert scale, anchored at 5 (“No change”), where 0 is “Much better” and 10 is “Much worse”, is used to assess change overall. The Patient’s Global Impression of Change is widely used to define clinically meaningful improvement in orthopedic and other pain studies. This instrument was first administered on the Day 3 assessment.

#### Impairment: Disabilities of the Arm, Shoulder, and Hand (DASH) or Lower Extremity Functional Scale (LEFS)

Separate validated questionnaires were used to assess upper extremity and lower extremity impairment.

The Disabilities of the Arm, Shoulder, and Hand (DASH) Outcome Measure was used for subjects with upper extremity envenomation [15,16]. The DASH is a 30-item self-administered questionnaire designed to assess impairment due to upper extremity injuries. The DASH has a standard period of recall of one week. Minor modifications were made to this tool at two time points. On the first assessment (just prior to hospital discharge), the tool was modified to collect subject recall of his or her baseline status prior to envenomation; in order to accomplish this, the recall period was adjusted to, “In the week prior to your snakebite…” On the Day 3 assessment, the tool was modified to collect information about the time period from hospital discharge to that assessment; the recall period was therefore adjusted to, “Since you were discharged from the hospital…” Although these modifications have not been validated, they were used in a previous trial with apparently good performance [10]. For the day 7, 14, 21, and 28 assessments, the standard recall period (“in the last week…”) was used. The range of possible scores is 0 – 100, and a higher score indicates more disability.

Subjects with lower extremity envenomation completed the Lower Extremity Functional Scale (LEFS), a widely-accepted, 20-item questionnaire that tests impairment due to lower extremity injuries [17]. The standard recall period for the LEFS is “today.” As with the DASH, the assessment made just prior to hospital discharge was modified to collect a pre-injury baseline (“In the day prior to your snakebite…”). The range of possible scores is 0 – 80; the minimal clinically important difference is 9 scale points, and a higher score indicates less disability.

#### Global Function: Patient-Specific Functional Scale (PSFS)

This is a three-item instrument, administered verbally, that is used to evaluate whether a health condition impacts a patient’s ability to perform activities that are important to him/her. On the initial assessment, the patient is asked to identify “up to three important activities that you are unable to do or are having difficulty with as a result of your (snakebite).” The patient then provides a rating for each item, on an 11-point ordinal scale ranging from 0 (“unable to perform activity”) to 10 (“able to perform activity at the same level as before the injury or problem”). During reassessments, the subject is prompted to re-rate the same three activities. The PSFS has been shown to be a sensitive, reliable, and responsive measure of impairment due to a wide variety of orthopedic conditions [18,19]. By design, the PSFS collects patient-oriented outcomes. Both 5-activity and 3-activity versions of this instrument have been described; because test performance is similar, we used the more parsimonious (3-activity) version. An average of the three “important activity” scores was used for calculations. This instrument was first administered on the Day 3 assessment.

#### Health-related quality of life: 36-item short-form health survey (SF-36^®^, v2 Acute version)

The Medical Outcomes Study 36-item Short-Form Health Survey, version 2 (SF-36^®^, v2 Acute version) is a self-administered questionnaire designed to evaluate overall health status [20]. Each item is an ordinal scale with 3 to 6 possible scores, weighted to produce overall score ranges of 0 – 100. Weighted averages are then used to summarize items within domains to create a Mental Component Scale (MCS) and Physical Component Scale (PCS). We utilized the acute version of the instrument, which has a period of recall of one week. As with the DASH and LEFS, during the time of discharge study visit we collected a recall-based estimation of the subject’s pre-envenomation baseline. Because of the look-back period, we did not collect this measure at the Day 3 assessment.

#### Ability to Work: Work Productivity and Ability Impairment: Special Health Problem, version 2 (WPAI: SHP)

This is a 6-item self-administered questionnaire designed to evaluate work limitation due to a specific health issue [21]. The WPAI: SHP is designed to capture both lost work time (“absenteeism”) and low work output (“presenteeism”). We used version 2 of this instrument, which consists of one yes/no question (“Are you currently employed (working for pay)?”), three questions about the number of hours of work / missed work, and one question that asks about productivity while at work. This final question collects responses on an 11-point ordinal scale, anchored at 0 (“Snakebite had no effect on my work”) and 10 (“Snakebite completely prevented me from working”). These five questions are used to calculate a percent of impairment from baseline work productivity. One additional question asks about ability to perform regular daily activities, other than work at a job, and ranged from 0 (“Snakebite had no effect on my daily activities”) to 10 (“Snakebite completely prevented me from doing my daily activities”). Although this question is collected on an ordinal scale, the results are communicated as a percent [22]. The WPAI: SHP was first administered on the Day 3 assessment.

#### Swelling: Figure-of-eight measurement

Objective measurements of swelling were obtained by using the combined circumference of the wrist/hand or foot/ankle, as appropriate for upper extremity or lower extremity envenomations. Investigators were trained to take figure-of-eight measurements using a ¼ inch (~6.5 mm) wide plastic retractable tape measure and standardized technique. Dressings more than 1 mm thick were removed prior to measurements. Measurements were recorded to the nearest 0.1 cm, and the median of three measurements was used for calculations. Figure-of-eight measurements have been shown to correlate well with limb swelling measurements obtained by more cumbersome methods, such as water volumetry, in both the upper and lower extremity [23-26]. Figure-of-eight measurements were performed prior to grip strength or walking speed testing.

#### Function: Grip strength or walking speed

To objectively measure hand function, patients with upper extremity envenomation performed grip strength measurements [27,28]. Three measurements of grip strength were performed using a JAMAR^®^ 5030 J1 Hydraulic Hand Dynamometer (Sammons Preston Roylan, Chicago, IL, USA), and the greatest value, measured in kilograms, was recorded.

To objectively measure lower extremity function, patients with lower extremity envenomation performed walking speed tests [29,30]. Beginning from standing, subjects walked 7.62 meters (25 feet) on indoor level ground as quickly, but safely, as possible without running. Two trials were performed, and the faster time was recorded. Subjects who could not complete the task without assistance or devices were not tested, and the maximum allowed time (180 seconds) was recorded.

#### Procedure for missed study assessments

If a subject missed a study visit, study personnel attempted to contact the subject by phone to arrange and reschedule a study visit within the permissible time window. If the patient refused or an in-person visit could not be arranged for some other reason, study personnel attempted to obtain all data for that visit by telephone, except figure-of-eight swelling measurements, grip strength, and/or walking speed. Subjects who missed 3 or more designated study visits were removed from subsequent participation the study, except for adverse event follow-up.

#### Safety assessments

At each visit, all adverse events (AEs) observed by the investigator or study personnel or reported by the patient were recorded in the medical record and entered into an adverse event reporting sheet. For each AE, the site investigator recorded a description of the event, seriousness, onset and stop dates for the event, severity, relationship to study participation, relationship to FabAV administration, action taken, and outcome. An AE was defined as serious using standard criteria, and both serious and non-serious AEs were reported to the FDA and IRBs in accordance with established requirements [31]. A secondary review of each reported AE was performed by the study safety monitor, who also reviewed the list of all medications used by subjects after enrollment and/or FabAV administration to look for AEs that may not have been reported by site investigators.

### Analysis

#### Data handling

Data were captured by trained study personnel using structured worksheets and entered into a Research Electronic Data Capture System (REDCap™) database.(version 5.0.015; REDCap Consortium and Vanderbilt University, Nashville, TN) [32]. Following data validation and error checks, the final database was exported to SAS (version 9.3; SAS Institute, Cary, NC) for analysis.

#### Sample size

Prior to this study, no evaluable data existed for limb recovery following crotaline envenomation. Formal power calculation was neither possible nor appropriate to the study’s intended purpose. Furthermore, logistical and budgetary considerations limited recruitment to a maximum of 11 study sites and half a snakebite “season”, leading to a target sample size of 20 evaluable subjects. Because of the observational and exploratory study design, no prespecified hypothesis testing and no study termination criteria for efficacy, futility, or safety were determined, and no interim analyses were planned. Adjustment for multiple statistical comparisons was not required because hypothesis testing was not performed.

#### Analysis plan

All analyses were performed by trained statisticians using SAS (version 9.3; SAS Institute, Cary, NC). Except where explicitly noted, all statistical analyses were planned *a priori.*

Hypothesis testing was neither planned nor performed. Because most data were either ordinal scale and/or skewed, data are summarized descriptively using median and range.

Recovery was defined as return to the patient’s own baseline on the DASH, LEFS, and SF-36^®^ MCS and PCS instruments, as assessed on the “look-back” questioning performed prior to hospital discharge, and to no impairment on the WPAI: SHP instrument. A pre-planned sensitivity analysis was performed defining recovery for the DASH as return to the US population mean score (10.1) [33]. For time-dependent variables (recovery on the DASH, LEFS, SF-36^®^ MCS/PCS, and WPAI: SHP instruments; return to work), time-to-event analytic methods were used to estimate median time to achieve the recovery endpoint. Return to work was assessed in the actual numbers of days to return while all other assessments are assessed at the day 3, 7, 14, 21, and 28 study assessments, when collected.

#### Subgroup analyses

For each assessment tool and time point, a primary analysis is reported for all subjects with data, and pre-planned subgroup analyses are presented for: (1) patients who did and did not receive FabAV; (2) patients with initially mild, moderate, and severe envenomation; and (3) patients with upper extremity and lower extremity envenomation. For the purposes of this study, envenomation severity was determined solely based on the number of major joints (wrist, elbow, ankle, knee) involved in limb swelling. Swelling that did not cross any major joints (e.g. confined to the hand) was defined as “mild.” Swelling that crossed one major joint (e.g. involving the hand and forearm) was defined as “moderate,” and swelling that crossed two major joints (e.g. from hand to upper arm) was defined as “severe.”

#### Handling of missing data; sensitivity analysis

The primary analysis was performed with the assumption that data are missing at random therefore missing data were not imputed for this primary analysis. Sensitivity analyses were conducted using linear interpolation to impute data points for which previous and subsequent observations were available, and Last Observation Carried Forward (LOCF) when these points were not available. When the data-point from the first assessment time-point was missing it was left as missing as neither LOCF nor linear interpolations are possible. Missing data for subjects who failed to complete 3 or more study visits were not imputed.

#### Correlations between outcome measures

In order to determine which outcome measures provided similar data, we calculated Spearman rank correlations between the numeric scores on each item.

### Human subjects protection

This study was conducted in accordance with the World Medical Association Declaration of Helsinki (1996) and current Good Clinical Practice in accordance with International Conference on Harmonisation standards. All study procedures were reviewed and approved by the Western Institutional Review Board (WIRB) and by the IRB responsible for each clinical site. The activities of the coordinating center were approved by the Colorado Multiple IRB (COMIRB). Written informed consent was obtained from all subjects prior to participation.

### Control of data and decision to publish

Duplicate copies of all audited data were maintained at the Rocky Mountain Poison and Drug Center and BTG International, Inc., and statisticians from both organizations had full access to all data. The statistical analysis plan was determined *a priori* and agreed-upon by both organizations. The trial was registered on www.ClinicalTrials.gov as protocol NCT01651299. A written commitment to publish study results within 18 months of study completion was established in the initial study protocol; BTG International retained a 60-day right to review the manuscript for proprietary information. The choice of journal was made jointly between the corresponding author and BTG International.

## Results

### Subject enrollment

**Figure 1:**
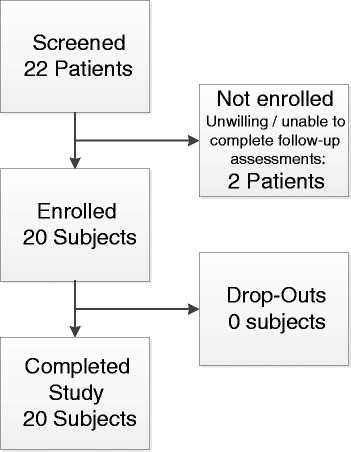
Participant Flow.

A total of 22 subjects were screened and 20 were enrolled in the study. Two potential subjects were excluded because they were unwilling or unable to complete follow-up assessments; no subjects were excluded because of distracting injuries or other comorbidities that would limit the validity of assessments. No subjects were lost to follow-up, though in one subject the Day 28 assessment was performed outside the allowed (+/− 3 day) window. Characteristics of study subjects are presented in Table 1, and subject enrollment flow appears in Figure 1. Half of subjects were male, most were non-Hispanic whites (80%), and the median age was 37 years. Upper extremity and lower extremity envenomations were evenly represented. Most subjects (80%) had moderate severity envenomation, and most (75%) received FabAV. Twelve subjects (60%) were enrolled at a single study site; four sites enrolled 1 – 3 subjects each, and six sites enrolled no subjects

**Table 1:** **Study subjects**

**Characteristic**	**Number of subjects (n, %)**
**Enrolled subjects**	**20 (100%)**
Completed 28 days of study participation	20 (100%)
**Sex**	
Male (n, %)	10 (50%)
**Age**	
Age (years) (median, range)	37 (19–76)
Aged ≥ 65 years	3 (15%)
**Race and ethnicity**	
White race	16 (80%)
Black or African American race	4 (20%)
Hispanic or Latino ethnicity	0 (0%)
**Study site**	
Duke University Medical Center (Durham, NC)	12 (60%)
East Carolina University/Vidant Medical Center (Greenville, NC)	3 (15%)
St. Joseph Regional Health Center (Bryan, TX)	3 (15%)
Scott and White Memorial Hospital (Temple, TX)	1 (5%)
University of Virginia (Charlottesville, VA)	1 (5%)
**Envenomation location**	
Upper extremity	10 (50%)
**Envenomation severity at enrollment**	
Mild	1 (5%)
Moderate	16 (80%)
Severe	3 (15%)
**Treatment**	
Treated with antivenom	15 (75%)
Total antivenom dose (vials) (among patients receiving) (median, range)	14 (4, 24)
**Duration of Hospitalization** (hours) (median, range)	35.5 (5, 49)

For the purposes of this study, envenomation severity was determined based on the number of major joints (wrist, elbow, ankle, knee) involved in limb swelling. Swelling that did not cross any major joints (e.g. confined to the hand) was defined as “mild.” Swelling that crossed one major joint (e.g. involving the hand and forearm) was defined as “moderate,” and swelling that crossed two major joints (e.g. from hand to upper arm) was defined as “severe”.

As expected, patients with more severe envenomation were more likely to receive FabAV therapy than patients with lesser severity envenomation: 3/3 (100%) of patients with severe envenomation received antivenom, compared with 12/16 (80%) of those with moderate severity envenomation. The one patient with mild severity envenomation did not receive FabAV. Imbalance was also seen between FabAV use and bite location: 9/10 (90%) of patients with lower extremity envenomation received FabAV, compared with 6/10 (60%) of those bitten on the upper extremity.

### Recovery from copperhead envenomation

#### Analgesic use

**Figure 2:**
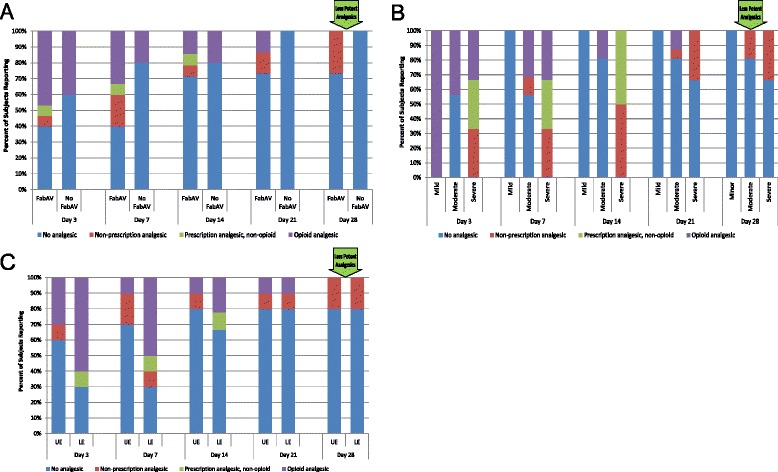
Analgesic Use: Subgroup Results. **A**: FabAV: Crotaline Polyvalent Immune Fab (ovine). Subgroup size: FabAV n = 15; No FabAV n = 5. **B**: Subgroup size: Mild n = 1, Moderate n = 16, Severe n = 3. **C**: Subgroup size: Upper extremity (UE) n = 10, Lower extremity (LE) n = 10.

Table 2 shows analgesic use for all time periods, and Figure 2A–C show analgesic use subgroup analyses based on FabAV therapy, envenomation severity, and envenomation site. Half of patients no longer required analgesics at the Day 7 assessment, and no patient required opioid analgesics by Day 28.

**Table 2:** **Analgesic use: Overall Study Results**

	**Day 3**		**Day 7**		**Day 14**		**Day 21**		**Day 28**	
	**n**	**%**	**n**	**%**	**n**	**%**	**n**	**%**	**n**	**%**
**No analgesic**	9/20	45	10/20	50	14/19	74	16/20	80	16/20	80
**Non-prescription analgesic**	1/20	5	3/20	15	1/19	5	2/20	10	4/20	20
**Prescription analgesic, non-opioid**	1/20	5	1/20	5	1/19	5	0/20	0	0/20	0
**Opioid analgesic**	9/20	45	6/20	30	3/19	16	2/20	10	0/20	0

Classification of the strongest type of medication used by study subjects to treat snakebite-related pain in the 24 hours prior to each scheduled study assessment. The day of envenomation is Day 0.

#### Recovery

**Figure 3:**
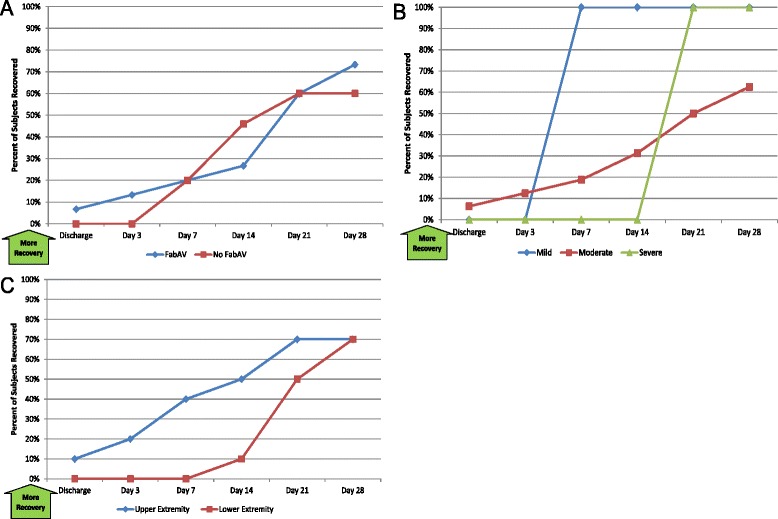
Patient Global Assessment of Recovery (PGAR): Subgroup Results. **A**: FabAV: Crotaline Polyvalent Immune Fab (ovine). Subgroup size: FabAV n = 15; No FabAV n = 5. **B**: Subgroup size: Mild n = 1, Moderate n = 16, Severe n = 3. **C**: Subgroup size: Upper extremity n = 10, Lower extremity n = 10.

Table 3 shows the patients’ global assessment of recovery for all time periods, and Figure 3A–C show these data by therapy, severity, and site. Fourteen of 20 subjects (70%) considered themselves to be fully recovered by the Day 28 assessment.

**Table 3:** **Patient’s Global Assessment of Recovery (PGAR): Overall Study Results**

	**Discharge**		**Day 3**		**Day 7**		**Day 14**		**Day 21**		**Day 28**	
	**n**	**%**	**n**	**%**	**n**	**%**	**n**	**%**	**n**	**%**	**n**	**%**
**Subjects reporting complete recovery**	1/20	5	2/20	10	4/20	20	6/20	30	12/20	60	14/20	70

Patients’ response to the question, “Have you completely recovered from your snakebite?” A larger number indicates a greater proportion of patients recovered. The day of envenomation is Day 0.

#### Self-reported pain and swelling

**Figure 4:**
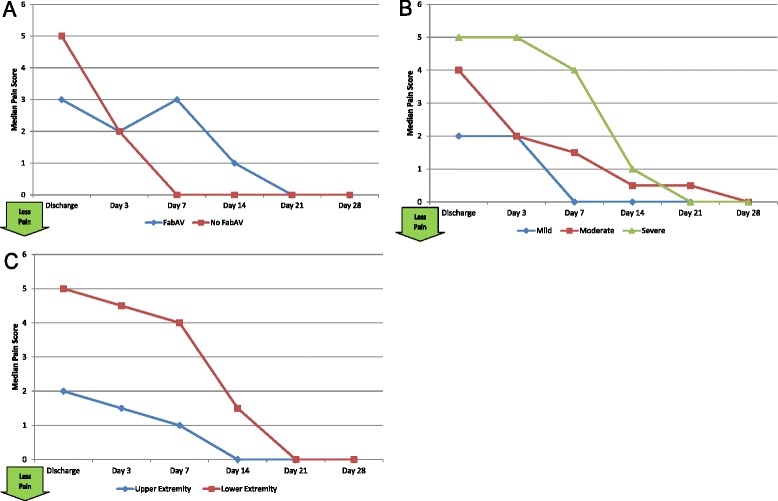
Numeric Pain Rating Scale (NPRS): Subgroup Results. **A**: FabAV: Crotaline Polyvalent Immune Fab (ovine). Subgroup size: FabAV n = 15; No FabAV n = 5. **B**: Subgroup size: Mild n = 1, Moderate n = 16, Severe n = 3. **C**: Subgroup size: Upper extremity n = 10, Lower extremity n = 10.

**Figure 5:**
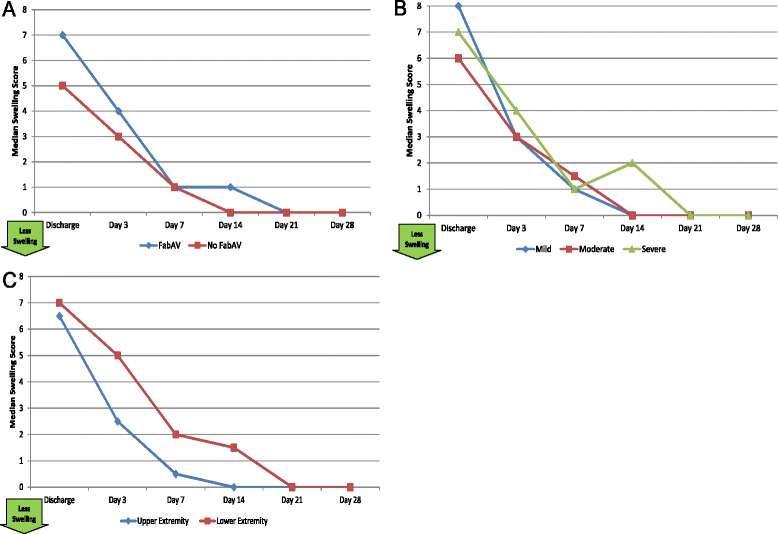
Numeric Swelling Scale (NSS): Subgroup Results. **A**: FabAV: Crotaline Polyvalent Immune Fab (ovine). Subgroup size: FabAV n = 15; No FabAV n = 5. **B**: Subgroup size: Mild n = 1, Moderate n = 16, Severe n = 3. **C**: Subgroup size: Upper extremity n = 10, Lower extremity n = 10.

Table 4 shows the patients’ self-reported pain at each study visit, and Figure 4A–C show subgroup results. In general, patients reported little pain at any time point, though patients with severe swelling and lower extremity envenomation seemed to fare worse on this measure for the first 2 weeks after envenomation. Table 5 and Figure 5A–C show patients’ self-reported swelling. Interestingly, there was little reported difference on this measure between subjects with mild, moderate, and severe initial swelling. All patients perceived swelling to be minimal by the Day 7 visit.

**Table 4:** **Numeric Pain Rating Scale (NPRS): Overall Study Results**

	**Discharge**		**Day 3**		**Day 7**		**Day 14**		**Day 21**		**Day 28**	
	**Median**	**Range**	**Median**	**Range**	**Median**	**Range**	**Median**	**Range**	**Median**	**Range**	**Median**	**Range**
**Score**	4	0, 10	2	0, 9	2.5	0, 8	0.5	0, 5	0	0, 4	0	0, 2

Subjects’ self-reported pain, ordinal scale ranging from 0 (“no pain”) to 10 (“worst possible pain”) [13]. Data based on 20 subjects. A larger number indicates more pain. The day of envenomation is Day 0.

**Table 5:** **Numeric Swelling Scale (NSS): Overall Study Results**

	**Discharge**		**Day 3**		**Day 7**		**Day 14**		**Day 21**		**Day 28**	
	**Median**	**Range**	**Median**	**Range**	**Median**	**Range**	**Median**	**Range**	**Median**	**Range**	**Median**	**Range**
**Score**	6.5	2, 10	3	0, 9	1	0, 9	0.5	0, 4	0	0, 3	0	0, 3

Subjects’ self-reported swelling, ordinal scale ranging from 0 (“no swelling”) to 10 (“very severe swelling”). A larger number represents more swelling. Data based on 20 subjects. The day of envenomation is Day 0.

#### Return to work, school, or usual activities of daily living (ADLs)

**Figure 6:**
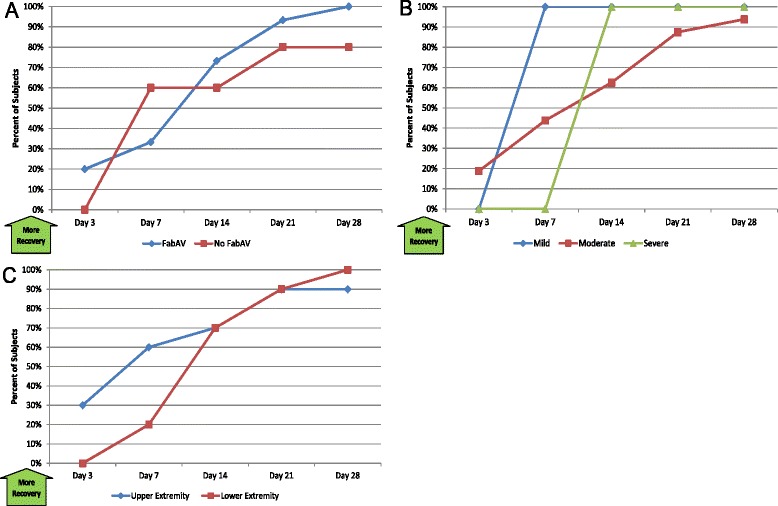
Return to Work, School, or Usual Activities of Daily Living (ADLs): Subgroup Results. **A**: FabAV: Crotaline Polyvalent Immune Fab (ovine). Subgroup size: FabAV n = 15; No FabAV n = 5. **B**: Subgroup size: Mild n = 1, Moderate n = 16, Severe n = 3. **C**: Subgroup size: Upper extremity n = 10, Lower extremity n = 10.

Table 6 and Figure 6A–C show patients’ self-reported return to full participation in their usual activities of daily living. The median time to return to full participation was 11 days (range: 2 to >28 days), with 1/20 subjects (5%) unable to return on Day 28.

**Table 6:** **Return to work, school, or usual Activities of Daily Living (ADLs): Overall Study Results**

	**Day 3**		**Day 7**		**Day 14**		**Day 21**		**Day 28**	
	**n**	**%**	**n**	**%**	**n**	**%**	**n**	**%**	**n**	**%**
**Subjects returned to usual activities**	3/20	15	8/20	40	14/20	70	18/20	90	19/20	95

Patients’ self-reported answer to the question, “Have you returned to full duties or participation at [state subject’s occupation]?” A larger number indicates a greater portion of patients who have returned to usual activities. The day of envenomation is Day 0.

#### Patient’s Global Impression of Change (PGIC)

**Figure 7:**
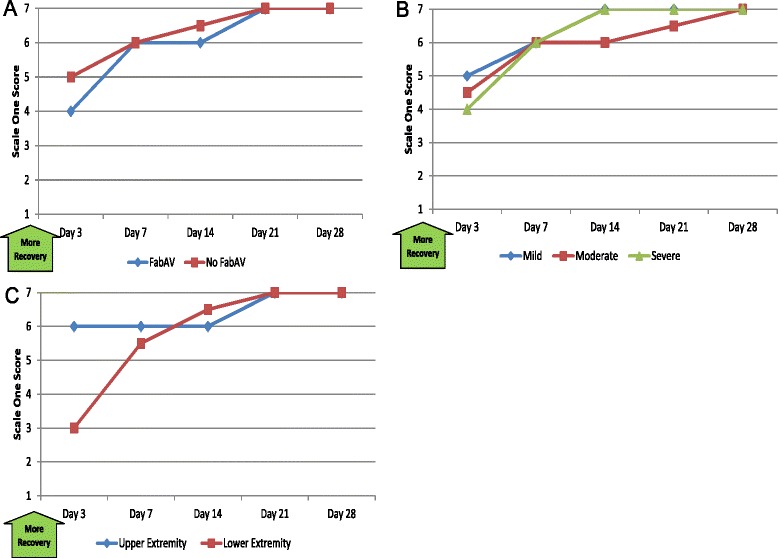
Patient’s Global Impression of Change (PGIC): Subgroup Results. **A**: FabAV: Crotaline Polyvalent Immune Fab (ovine). Subgroup size: FabAV n = 15; No FabAV n = 5. Missing data from one FabAV subject on Day 3 and one No FabAV subject on Day 14. **B**: Subgroup size: Mild n = 1, Moderate n = 16, Severe n = 3. Missing data from 1 Severe subject on Day 3 and one Moderate subject on Day 14. **C**: Subgroup size: Upper extremity n = 10, Lower extremity n = 10. Missing data from one Lower Extremity subject on Day 3 and one Upper Extremity subject on Day 14.

Table 7 and Figure 7A–C show the patients’ self-reported impression of change. Patients’ perceived improvement between initiation of care and the Day 3 assessment, and most patients perceived a great deal of improvement by Day 14. Subgroup results on the PGIC Scale Two score (not shown) were similar to those on the Scale One score. One subject was provided an anomalous result on the Scale Two score on the Day 28 assessment. Despite improving scores on the PSFS (improvement from 0 to 7.33), LEFS (5 to 65), SF-36 PCS (22.5 to 51.4), and PGIC Scale one score (2 to 6), he reported being “much worse” (score 9) on the PGIC Scale Two score. This is also inconsistent with his prior responses on PGIC Scale Two question (serial scores of 9, 4, 4, and 2), which showed improvement consistent with that reported on the other instruments.

**Table 7:** **Patient’s Global Impression of Change (PGIC): Overall Study Results**

	**Day 3**		**Day 7**		**Day 14**		**Day 21**		**Day 28**	
	**Median**	**Range**	**Median**	**Range**	**Median**	**Range**	**Median**	**Range**	**Median**	**Range**
**Scale One score**	5	1, 7	6	2, 7	6	2, 7	7	1, 7	7	1, 7
**Scale Two score**	4	0, 9	2	0, 4	1	0, 6	0	0, 5	0	0, 9

Both scores assess the patient’s self-report of change, “since beginning treatment at this clinic” [14]. Scale one is a 7-item Likert scale, ranging from 1 (“No change (or condition has gotten worse)”) to 7 (“A great deal better, and a considerable improvement that has made all the difference”). Scale two is an 11-item Likert scale, ranging from 0 (“Much better”) to 10 (“Much worse”). Therefore, higher scores on scale one indicate more recovery and higher scores on scale two indicate less recovery. Data based on 20 subjects except for Day 3 (n =19). The day of envenomation is Day 0. One subject provided an anomalous result to the Scale Two score on Day 28 (see text).

#### Impairment: DASH and LEFS

**Figure 8:**
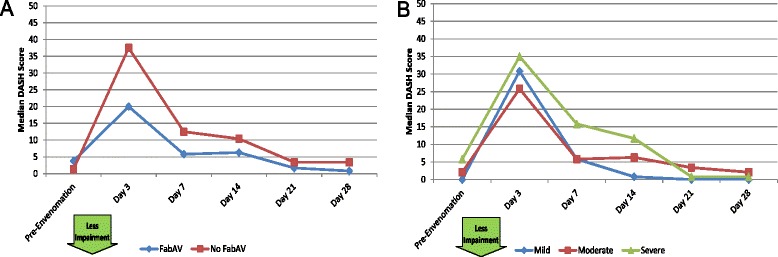
Disabilities of the Arm, Shoulder, and Hand (DASH) Scores: Subgroup Results. **A**: FabAV: Crotaline Polyvalent Immune Fab (ovine). Subgroup size: FabAV n = 6; No FabAV n = 4. **B**: Subgroup size: Mild n = 1; Moderate n = 8; Severe n = 1.

Table 8 and Figure 8A–B show impairment for patients with upper extremity envenomation. Patients reported marked impairment at the Day 3 assessment and marked improvement thereafter. The median time for a subject to return to his/her pre-envenomation baseline was 21 days (range: 3 to >28 days), and at the end of the 28-day study period, 2/10 (20%) of patients had not recovered to their own baseline. Data for the US population are available for the DASH, and analysis showed a median time of 7 days (range: 3 to >28 days) to recover to the US population mean score on the DASH instrument.

**Table 8:** **Disabilities of the Arm, Shoulder, and Hand (DASH) score: Overall Study Results**

	**Pre-Envenomation**		**Day 3**		**Day 7**		**Day 14**		**Day 21**		**Day 28**	
	**Median**	**Range**	**Median**	**Range**	**Median**	**Range**	**Median**	**Range**	**Median**	**Range**	**Median**	**Range**
**Score**	2.1	0.0, 11.7	28.3	0.0, 71.7	7.1	0.0, 63.3	6.3	0.0, 30.8	1.7	0.0, 20.8	0.8	0.0, 12.5

Score on the Disabilities of the Arm, Shoulder, and Hand (DASH) outcome measure [15,16]. The range of possible scores is 0 – 100. Higher scores indicate more disability. Evaluation limited to subjects with upper extremity envenomation (n = 10). The day of envenomation is Day 0. Use of “look back” questioning to establish pre-injury baseline has not been previously validated for the DASH.

**Figure 9:**
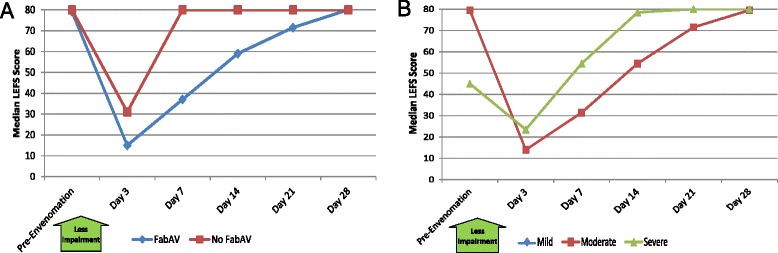
Lower Extremity Functional Scale (LEFS) Scores: Subgroup Results. **A**: FabAV: Crotaline Polyvalent Immune Fab (ovine). Subgroup size: FabAV n = 9; No FabAV n = 1. Missing data from one FabAV subject on Day 21. **B**: Subgroup size: Mild n = 0, Moderate n = 8, Severe n = 2. Missing data from one Severe subject on Day 21.

Patients with lower extremity envenomation (Table 9 and Figure 9A–B) showed more gradual recovery, with a median time to recover to personal baseline of 14 days (range: 3 to >28 days). At the end of the 28-day study period, 2/10 (20%) of patients had not recovered to their own baseline. Normative data about the US population are not available for the LEFS.

**Table 9:** **Lower Extremity Functional Scale (LEFS): Overall Study Results**

	**Pre-Envenomation**		**Day 3**		**Day 7**		**Day 14**		**Day 21**		**Day 28**	
	**Median**	**Range**	**Median**	**Range**	**Median**	**Range**	**Median**	**Range**	**Median**	**Range**	**Median**	**Range**
**Score**	79	12, 80	17	5, 31	38	8, 80	67.5	23, 80	72	60, 80	80	65, 80

Score on the Lower Extremity Functional Scale (LEFS) outcome measure [17]. The range of possible scores is 0 – 80. Higher scores indicate less disability. Evaluation limited to subjects with lower extremity envenomation (n = 10) except for Day 21 (n = 9). The day of envenomation is Day 0. Use of “look back” questioning to establish pre-injury baseline has not been previously validated for the LEFS.

#### Function in activities important to the patient (PSFS)

**Figure 10:**
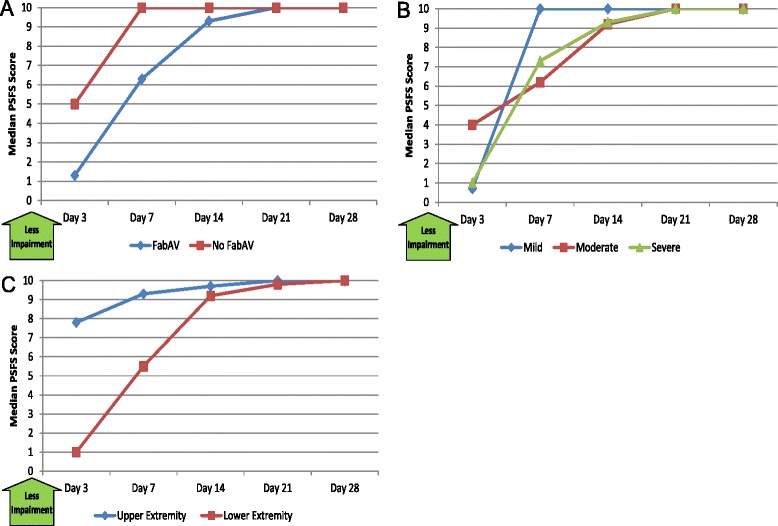
Patient-Specific Functional Scale (PSFS): Subgroup Results. **A**: FabAV: Crotaline Polyvalent Immune Fab (ovine). Subgroup size: FabAV n = 15; No FabAV n = 5. **B**: Subgroup size: Mild n = 1, Moderate n = 16, Severe n = 3. **C**: Subgroup size: Upper extremity n = 10, Lower extremity n = 10.

Table 10 and Figure 10A–C show the patients’ reported ability to participate in activities that are self- identified as important by the subject. Patients reported a great deal of limitation through Day 7 and little limitation by 21 days after envenomation.

**Table 10:** **Patient-Specific Functional Scale (PSFS): Overall Study Results**

	**Day 3**		**Day 7**		**Day 14**		**Day 21**		**Day 28**	
	**Median**	**Range**	**Median**	**Range**	**Median**	**Range**	**Median**	**Range**	**Median**	**Range**
**Score**	2.2	0, 10	6.8	0, 10	9.3	0, 10	10.0	0, 10	10.0	0, 10

Score on the Patient-Specific Functional Scale outcome measure [17]. The average of up to 3 specific activity scores was recorded, and the range of possible scores is 0 – 10. Higher scores indicate less impairment. Data based on 20 subjects. The day of envenomation is Day 0.

#### Health-related quality of life (SF-36^®^ v2, Acute version)

**Figure 11:**
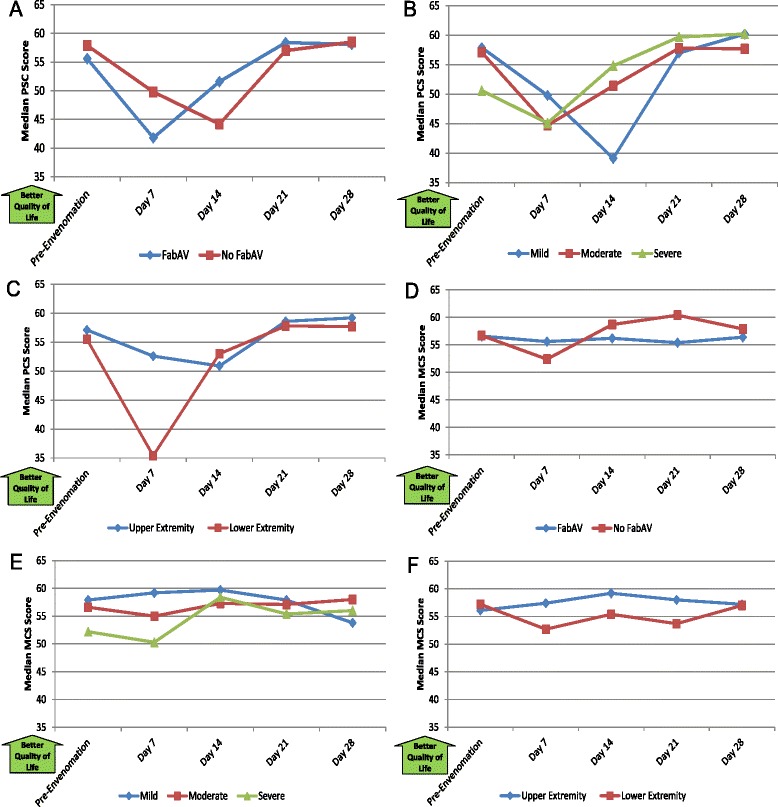
Quality of Life, 36-item Short Form Health Survey (SF-36^®^, v2 Acute version) Physical Component Scale and Mental Component Scale Scores: Subgroup Results. **A**: FabAV: Crotaline Polyvalent Immune Fab (ovine). Subgroup size: FabAV n = 15; No FabAV n = 5. **B**: Subgroup size: Mild n = 1, Moderate n = 16, Severe n = 3. **C**: Subgroup size: Upper extremity n = 10, Lower extremity n = 10. **D**: FabAV: Crotaline Polyvalent Immune Fab (ovine). Subgroup size: FabAV n = 15; No FabAV n = 5. **E**: Subgroup size: Mild n = 1, Moderate n = 16, Severe n = 3. **F**: Subgroup size: Upper extremity n = 10, Lower extremity n = 10.

Table 11 and Figure 11A–F show the patients’ self-reported quality of life. As expected, the SF-36^®^ PCS scores showed more change over the 28-day study than the MCS scores. Subjects reported decrements in physical quality of life for the first 2 weeks after envenomation, after which most subjects rated their quality of life as similar to before the envenomation occurred. The median time to return to baseline was 21 days (range: 7 to >28 days) for the PCS and 10.5 days (range: 7 to >28 days) for the MCS. At the Day 28 assessment, 7/20 patients (35%) reported PCS scores and 9/20 patients (45%) reported MCS scores that were worse than their pre-envenomation baselines.

**Table 11:** **36-Item Short Form Health Survey (SF-36** ^**®**^ **, v2 Acute version): Overall Study Results**

	**Pre-Envenomation**		**Day 7**		**Day 14**		**Day 21**		**Day 28**	
	**Median**	**Range**	**Median**	**Range**	**Median**	**Range**	**Median**	**Range**	**Median**	**Range**
**SF-36 Physical Component Summary score**	56.0	23.9, 60.2	45.1	18.3, 60.5	51.4	22.8, 60.5	58.3	38.0, 60.3	58.3	47.7, 60.4
**SF-36 Mental Component Summary score**	56.6	43.5, 68.8	55.0	28.9, 65.8	58.3	43.0, 63.8	56.5	39.5, 64.4	57.2	47.6, 61.9

Scores on the SF-36^®^, v2 (Acute Version) outcome measure Physical Component Summary and Mental Component Summary measures [20]. The range of possible scores on all measures is 0–100. Higher scores indicate better quality of life. Data based on 20 subjects. The day of envenomation is Day 0. Use of “look back” questioning to establish pre-injury baseline has not been previously validated for the SF-36.

#### Work productivity and daily activity impairment (WPAI: SHP, version 2)

Evaluation of responses to the Work Productivity Impairment items on the WPAI: SHP instrument showed uninterpretable results. Of the 20 study subjects, 7 reported that they were not employed at any of the assessment time points, and 2 additional subjects appeared to have lost, gained, and/or regained employment during the course of the study. Of the remaining 11 subjects, we were unable to calculate a work productivity impairment score at one or more time points for 4 subjects due to missing responses or a sum of 0 scheduled work hours [hours actually worked + hours missed due to snakebite + hours missed for other reasons]. Of the remaining 7 study subjects, 6 subjects provided data showing a sum of scheduled work hours that varied more than 100% between the assessments. Therefore, work productivity impairment is not reported.

**Figure 12:**
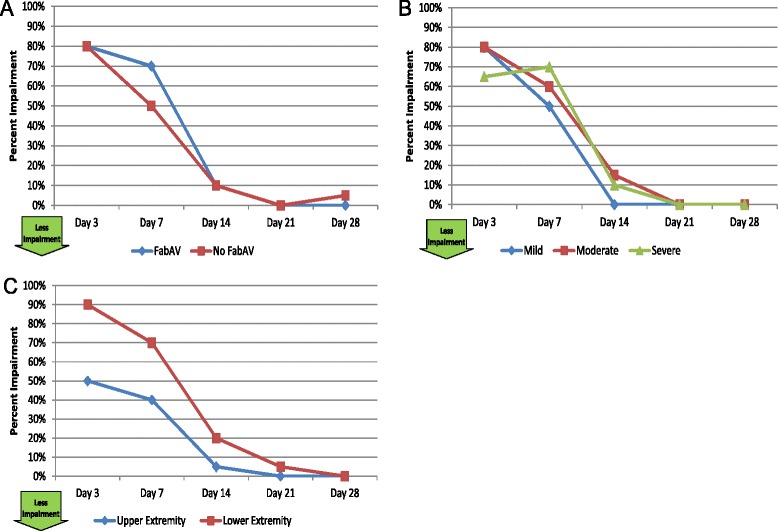
Daily Activities Impairment: Subgroup Results. **A**: FabAV: Crotaline Polyvalent Immune Fab (ovine). Subgroup size: FabAV n = 15; No FabAV n = 5. **B**: Subgroup size: Mild n = 1, Moderate n = 16, Severe n = 3. **C**: Subgroup size: Upper extremity n = 10, Lower extremity n = 10.

Table 12 and Figure 12A–C show impairment of daily activities outside of work for all 20 subjects. The median time to return to full participation in daily activities outside work was 21 days (range: 3 to >28 days), and 5/20 subjects (25%) reported less than full participation in non-work activities 28 days after envenomation.

**Table 12:** **Impairment at daily activities outside of work: Overall Study Results**

	**Day 3**		**Day 7**		**Day 14**		**Day 21**		**Day 28**	
	**Median**	**Range**	**Median**	**Range**	**Median**	**Range**	**Median**	**Range**	**Median**	**Range**
**Percent Daily Activities Impairment**	80	0, 100	60	0, 100	10	0, 80	0	0, 40	0	0, 30

Percent impairment of regular daily activities other than work is calculated by taking self-reported impairment on item 6 of the Work Productivity and Ability Impairment: Special Health Problem instrument. This is an 11-item ordinal scale, ranging from 0 “(Snakebite had no effect on my daily activities”) to 10 (“Snakebite completely prevented me from doing my daily activities”), multiplied by 10. Higher scores indicate more impairment. The day of envenomation is Day 0.

#### Objectively-measured swelling

**Figure 13:**
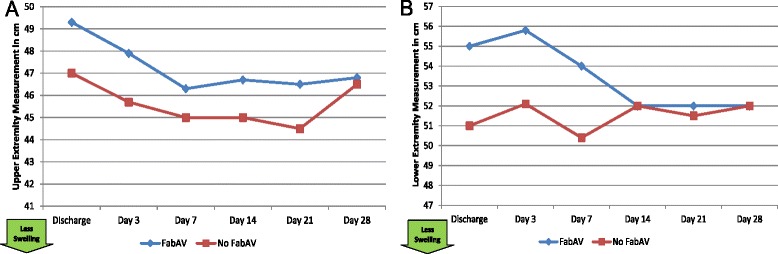
Figure-of-Eight Swelling Measurements: Subgroup Results. **A**: Upper Extremity. FabAV: Crotaline Polyvalent Immune Fab (ovine). Subgroup size: FabAV n = 6; No FabAV n = 4. Missing data from one FabAV subject on Days 7 and 14. **B**: Lower Extremity. FabAV: Crotaline Polyvalent Immune Fab (ovine). Subgroup size: FabAV n = 9; No FabAV n = 1. Missing data from one FabAV subject on Day 3.

Despite promising results from the use of figure-of-eight measurements in other orthopedic conditions, these measurements did not appear to track with other measures of recovery (Table 13; Figure 13A–B). It is unclear whether this is because swelling resolved prior to the Day 3 measurement, swelling did not involve the wrist/hand or ankle/foot, swelling was effectively managed by other means (e.g. wrapping, elevation), or the measurements were not consistent and accurate.

**Table 13:** **Figure-of-eight swelling measurements: Overall Study Results**

	**Discharge**		**Day 3**		**Day 7**		**Day 14**		**Day 21**		**Day 28**	
	**Median**	**Range**	**Median**	**Range**	**Median**	**Range**	**Median**	**Range**	**Median**	**Range**	**Median**	**Range**
**Upper extremity measurement (cm)**	47.0	38.0, 51.8	46.7	38.0, 51.6	45.0	37.5, 50.5	45.0	37.5, 51.5	45.5	37.5, 50.0	46.5	37.2, 50.3
**Upper extremity, difference from measurement at hospital discharge (cm)**	--	--	−0.9	−3.5, 0.0	−2.0	−3.7, 0.0	−2.0	−3.6, +0.1	−2.8	−4.0, 0.5	−1.3	−3.8, +1.0
**Lower extremity measurement (cm)**	54.5	49.3, 61.0	55.8	49.0, 61.1	53.9	46.5, 60.8	52.0	47.0, 60.2	51.8	46.7, 56.5	52.0	46.4, 57.0
**Lower extremity, difference from measurement at hospital discharge (cm)**	--	--	+0.5	−2.5, +3.5	−1.3	−2.8, +1.4	−2.2	−3.5, +1.0	−2.7	−4.5, +0.5	−2.7	−4.5, +1.0

Measurements around the wrist/hand or ankle/foot [23-26]. Upper extremity measurements based on 10 subjects with upper extremity envenomation except for the Day 7 and Day 14 measurements (n-9). Lower extremity measurements based on 10 subjects with lower extremity envenomation except for the Day 3 measurement (n = 9). Higher numbers indicate more swelling. The day of envenomation is Day 0.

#### Objectively-measured function

**Figure 14:**
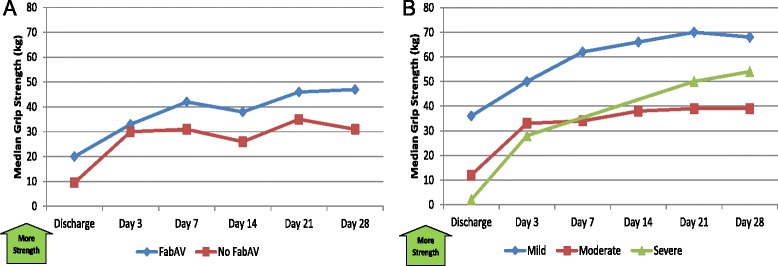
Grip Strength: Subgroup Results. **A**: FabAV: Crotaline Polyvalent Immune Fab (ovine). Subgroup size: FabAV n = 6; No FabAV n = 4. Missing data from one FabAV subject on Days 7 and 14. **B**: Subgroup size: Mild n = 1; Moderate n = 8; Severe n = 1. Missing data from the Severe subject on Days 7 and 14.

Among patients bitten on the hand or forearm, median grip strength increased 217% from the discharge to Day 14 assessments, then plateaued (Table 14; Figure 14A–B).

**Table 14:** **Grip strength: Overall Study Results**

	**Discharge**		**Day 3**		**Day 7**		**Day 14**		**Day 21**		**Day 28**	
	**Median**	**Range**	**Median**	**Range**	**Median**	**Range**	**Median**	**Range**	**Median**	**Range**	**Median**	**Range**
**Strength (kg)**	12	UTP, 40	33	2, 57	34	8, 62	38	11, 66	46	12, 70	40	18, 68
**Number of subjects unable to complete test**	1/10 (10%)		0/10 (0%)		0/9 (0%)		0/9 (0%)		0/10 (0%)		0/10 (0%)	

Grip strength, measured in kilograms [27,28]. UTP: Unable to perform test. Data from 10 subjects with upper extremity envenomation except for the Day 7 and Day 14 measurements (n = 9). Higher numbers indicate more strength. The day of envenomation is Day 0.

**Figure 15:**
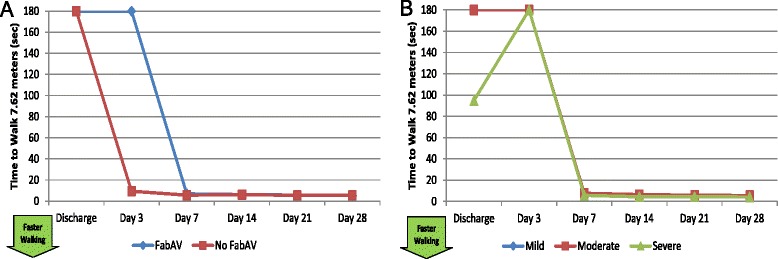
Walking Speed: Subgroup Results. **A**: FabAV: Crotaline Polyvalent Immune Fab (ovine). Subgroup size: FabAV n = 9; No FabAV n = 1. **B**: Subgroup size: Mild n = 0, Moderate n = 8, Severe n = 2.

Most patients bitten on the leg or ankle could not walk without assistance through at least the first three days after injury. Most were walking well by Day 7. Because the only mildly- envenomated subject in this study was bitten on the arm, no mildly-envenomated subjects completed the walking speed assessment (Table 15; Figure 15A–B).

**Table 15:** **Walking speed: Overall Study Results**

	**Discharge**		**Day 3**		**Day 7**		**Day 14**		**Day 21**		**Day 28**	
	**Median**	**Range**	**Median**	**Range**	**Median**	**Range**	**Median**	**Range**	**Median**	**Range**	**Median**	**Range**
**Time to walk 7.62 meters (sec)**	UTP	7.1, UTP	UTP	6.8, UTP	6.7	4.9, UTP	6.2	4.1, 11.4	5.5	4.3, 7.2	5.3	4.0, 6.2
**Number of subjects unable to complete test**	7/10 (70%)		6/9 (66%)		2/10 (20%)		0/10 (0%)		0/10 (0%)		0/10 (0%)	

Time to walk 7.62 meters (25 feet) on indoor level ground, in seconds [29,30]. UTP: Unable to perform test (unable to safely walk 7.62 meters without assistance devices in 180 seconds or less). Data from 10 subjects with lower extremity envenomations except for the Day 3 measurements (n = 9). Higher numbers indicate slower ambulation. The maximum amount of time allowed for the task is 180 seconds, and subjects who were unable to complete the task were scored as 180 seconds. The day of envenomation is Day 0.

#### Correlation between study measures

Table 16 provides data about the correlation between different outcome measures at Day 14. Out of 62 comparisons, 55 had correlation coefficients in the expected direction (i.e. both tests suggested improvement or worsening), and 6 of the 7 of the remaining correlations were weak (ρ < 0.15). There was a moderate correlation (ρ = 0.65) between grip strength and figure-of-8 swelling measurement of the wrist that suggested the higher the grip strength the more swelling. We theorize that this is due to the fact that figure-of-8 measures both the patient’s size and superimposed swelling. Given that there was little change in the figure-of-8 measurements over time and figure-of-8 measurements were very weakly correlated with self-reported swelling (ρ = 0.08), the most likely explanation is confounding based on the fact that larger patients may be stronger than smaller patients.

**Table 16:** **Correlation between assessment items (Day 14 time point; absolute values shown)**

**Variable**	**Walking Speed**	**Grip Strength**	**Figure- of-8 Swelling**	**PSFS**	**WPAI:SHP Daily Activities Impairment**	**DASH**	**LEFS**	**PGIC**	**NSS**	**NPRS**	**SF-36v2 MCS**	**SF-36v2 PCS**
**Walking Speed**	1	n/a	0.66	0.72	0.76	n/a	0.71	0.84	0.25	0.75	0.42	0.64
**Grip Strength**	--	1	0.65*	0.84	0.73	0.81	n/a	0.86	0.58	0.70	0.26	0.29
**Figure-of-8 Swelling**			1	0.06*	0.33	0.62	0.48	0.14*	0.08	0.08	0.37	0.08
**PSFS**				1	0.72	0.95	0.83	0.79	0.61	0.93	0.78	0.67
**WPAI:SHP Daily Activities Impairment**					1	0.72	0.80	0.55	0.61	0.80	0.21	0.59
**DASH**						1	n/a	0.64	0.55	0.88	0.03*	0.73
**LEFS**							1	0.92	0.65	0.93	0.15	0.88
**PGIC**								1	0.43	0.68	0.01*	0.52
**NSS**									1	0.75	0.08*	0.47
**NPRS**										1	0.02	0.71
**SF-36v2 MCS**											1	0.12*
**SF-36v2 PCS**												1

PSFS: Patient-Specific Functional Scale score [18,19]; WPAI:SHP: Work Productivity and Ability Impairment: Special Health Problem instrument, version 2 [21]; DASH: Disorders of the Arm, Shoulder, and Hand outcome measure [15,16]; PGIC: Patient’s Global Impression of Change, Scale 1 score [14]; LEFS: Lower Extremity Functional Scale [17]; NSS: Numeric Swelling Scale; NPRS: Numeric Pain Rating Scale; SF-36^®^ MCS and PCS: Mental and Physical Component Scores on the Medical Outcomes Study 36-item Short Form Health Survey version 2 (SF-36^®^, v2) Acute Version instrument; n/a: not applicable; nd: no data or insufficient data to make this calculation. Because increasing values indicate improvement in some outcome measures and decrement in others, absolute values for correlations are shown. Results of the PGIC Scale 2 score are similar to the Scale 1 score (data not shown).

*Correlation is in the opposite direction from what is expected based on the design of the test (i.e. one test suggests worsening and the other suggests improvement).

The Patient-Specific Functional Scale and the WPAI: SHP Daily Activities Impairment question were moderately to highly correlated (ρ ≥ 0.6) with each other and with a broad range of other study instruments. Two single-item questions, the Numeric Pain Rating Scale and the Patient’s Global Impression of Change, were moderately to highly correlated with more complex methods of assessing limb impairment, including the 30-item DASH and 12-item LEFS instruments. The 11-item QuickDASH, a subset of the DASH instrument, was highly correlated with the DASH (Pearson’s correlation coefficient range 0.81 – 0.99 at different time points).

The best patient-reported instruments, such as PSFS, DASH, and LEFS, had at least moderately strong correlation (Spearman’s ρ 0.71 – 0.84) with the objective measurements of function, grip strength and the ability to walk 25 feet/7.62 meters without assistance.

In this study, several simple questions (PSFS, WPAI: SHP Daily Activities impairment question, Numeric Pain Rating Scale, Patient’s Global Impression of Change) and objective tests (grip strength, 25 foot walking test) provided consistent information, suggesting that these parsimonious tools may be valid and practical ways to assess limb recovery in snakebite victims.

#### Safety

Ten adverse events (AEs) were reported by 7 subjects during the time of subjects’ participation in the study (from the time of consent through completion of the Day 28 assessment.) None were rated as “serious” or judged to be related to study participation.

Four patients (27% of those receiving FabAV) had a total of 5 acute hypersensitivity reactions to FabAV. Three of these reactions occurred prior to study enrollment and 2 during study participation. One patient had a medically significant reaction, consisting of wheezing, dyspnea, hypotension, and rash, which began shortly after the FabAV infusion rate was increased from 20 mL/hour to 250 mL/hour. FabAV was discontinued, and the patient received oxygen, intravenous fluids, methylprednisolone, diphenhydramine, and famotidine. His blood pressure and respiratory distress improved, as did the swelling in his envenomated limb. He was discharged from the hospital two days later without incident. Another subject had 2 distinct episodes of acute hypersensitivity reaction to FabAV. This patient developed an urticarial rash during the initial FabAV infusion which improved with diphenhydramine. She was able to complete the initial FabAV dose without further incident. Several hours later she received a second dose of FabAV for progressive limb swelling, and experienced recurrence of her rash. This responded well to additional diphenhydramine, and she completed her second dose of FabAV without difficulty. At no time did she develop wheezing or hypotension, and her clinical course was otherwise unremarkable. Two additional subjects had mild urticarial reactions that responded to antihistamine administration.

## Discussion

Copperhead envenomation commonly causes pain and swelling in the envenomated limb that interferes with the patient’s quality of life and ability to function in work and leisure activities. In this first study to examine the natural history of this condition, patients typically had pain and limitations lasting about 2 weeks after envenomation. Most subjects, but not all, were fully recovered by 1 month after envenomation. Recovery may have been slower for patients with lower extremity envenomation.

A very simple patient-reported outcome measure, the PSFS, was both highly correlated with more complex assessment tools and very responsive to changes in patient condition over time. The use of patient-reported outcome measures has been advocated by the FDA and others as a preferred way to assess outcomes in clinical trials [34]. Within the limitations of its size and design, this study suggests that parsimonious and patient-centered tools are well-suited to study recovery from snake envenomation. Two easily-obtained measurements of function, grip strength and the ability to walk 25 feet/7.62 meters without assistance also performed well in this study.

This study is limited by its observational study design and small sample size. In particular, 75% of the subjects in this study received FabAV, and the patients managed with FabAV tended to have a more severe envenomation. Several factors, including small sample size and the lack of a standardized assessment of severity at the time a decision about FabAV administration was made, precluded the use of propensity-adjustment, propensity-matching, or stratified analytic methods to adjust for confounding by indication. This study was designed to be exploratory rather than hypothesis-testing; given the lack of formal statistical testing for significant differences or adjustment for multiple measures, no strong conclusions about subgroups can be supported. This study cannot be used to prove or disprove a clinical benefit associated with FabAV administration.

The problem of small sample size is particularly important given the large variability observed in some measures and the fact that skewed and ordinal data required the use of nonparametric statistical tests. These reasons alone should cause the reader to interpret the specific results with caution, as the estimate of effect for most measures is imprecise. An additional important limitation is that 60% of subjects came from a single institution, where both the local fauna and treatment resources may produce different outcomes than experienced elsewhere. If a difference in clinical disease spectrum is present in envenomations by copperheads from different subspecies (e.g. *A. c. contortrix* vs. *A. c. mokasin*) or regions (e.g. North Carolina vs. Texas), the generalizability of these results would be threatened.

Pain is an inherently subjective experience. In addition to the numeric pain rating score, pain is directly assessed in the DASH (2/30 items) and the SF-36 (2/36 items). Pain also contributes to limitations in role function (measured in the PSFS, DASH, LEFS, and WPAI: SHP), physical function (grip strength, walking speed), quality of life (SF-36), and analgesic use. To the extent that a subject’s perception of pain is influenced by psychosocial factors, medical and psychiatric comorbidities, prior medication and other substance use, and personality, these instruments may be measuring more than just the effects of venom on human tissue. Acknowledging these limits, pain and its effect on function are important, patient-centered outcomes and are important to consider in this context.

## Conclusions

In this population of patients envenomated by copperheads, most of whom received antivenom, pain, swelling, and disability lasted 7 – 21 days in most patients. Several tools appear to be responsive and useful in studying recovery from pit viper envenomation. These include the DASH, LEFS, PSFS, SF-36^®^ PCS, WPAI: SHP Daily Activities Impairment component, and structured self-reports of pain, swelling, analgesic use, recovery, and return to usual activities.

## Abbreviations

ADLs: Activities of Daily Living
AE: Adverse Event
DASH: Disorders of the Arm, Shoulder, and Hand
ED: Emergency Department
FabAV: Fab antivenom (Crotalidae Polyvalent Immune Fab (Ovine))
IRB: Institutional Review Board
LE: Lower Extremity
LEFS: Lower Extremity Functional Score
LOCF: Last Observation Carried Forward
MCS: Mental Component summary score (of the SF-36^®^, v2 Acute version)
PCS: Physical Component summary score (of the SF-36^®^, v2 Acute version)
PGIC: Patient’s Global Impression of Change
PSFS: Patient-Specific Functional Scale
REDCap™: Research Electronic Data Capture
SF-36^®^: v2, Medical Outcomes Study 36-item Short-Form Health Survey, version 2
UE: Upper Extremity
US: United States
WPAI: SHPv2: Work Productivity and Ability Impairment: Special Health Problem, version 2

## References

[1] O'Neil ME, Mack KA, Gilchrist J (2007). Epidemiology of non-canine bite and sting injuries treated in U.S. Emergency Departments, 2001–2004. Public Health Rep.

[2] Bronstein AC, Spyker DA, Cantilena LR, Rumack BH, Dart RC (2012). 2011 Annual report of the American Association of Poison Control Centers' National Poison Data System (NPDS): 29th Annual Report. Clin Toxicol (Phila).

[3] Dart RC, Seifert SA, Boyer LV, Clark RF, Hall E, McKinney P (2001). A randomized multicenter trial of crotalinae polyvalent immune Fab (ovine) antivenom for the treatment for crotaline snakebite in the United States. Arch Intern Med.

[4] Dart RC, Seifert SA, Carroll L, Clark RF, Hall E, Boyer-Hassen LV (1997). Affinity-purified, mixed monospecific crotalid antivenom ovine Fab for the treatment of crotalid venom poisoning. Ann Emerg Med.

[5] Spiller HA, Bosse GM, Ryan ML (2010). Use of antivenom for snakebites reported to United States poison centers. Am J Emerg Med.

[6] Lavonas EJ, Gerardo CJ, O'Malley G, Arnold TC, Bush SP, Banner W (2004). Initial experience with Crotalidae polyvalent immune Fab (ovine) antivenom in the treatment of copperhead snakebite. Ann Emerg Med.

[7] Lavonas EJ, Kokko J, Schaeffer TH, Mlynarchek SL, Bogdan GM, Dart RC (2011). Short-term outcomes after Fab antivenom therapy for severe crotaline snakebite. Ann Emerg Med.

[8] Yin S, Kokko J, Lavonas E, Mlynarchek S, Bogdan G, Schaeffer T (2011). Factors associated with difficulty achieving initial control with crotalidae polyvalent immune fab antivenom in snakebite patients. Acad Emerg Med.

[9] Gerardo CJ, Evans CS, Kuchibhatla M, Drake WG, Mando-Vandrick JD, Yen M (2013). Time to Antivenom Administration in Snakebite. Ann Emerg Med.

[10] Lavonas EJ, Kerns WP, Gerardo CJ, Richardson W, Whitlow K, Berkoff DJ (2008). 328: Long-Term Limb Function Outcomes Following Copperhead Snakebite. Ann Emerg Med.

[11] Thorson A, Lavonas EJ, Rouse AM, Kerns WP (2003). Copperhead envenomations in the Carolinas. J Toxicol Clin Toxicol.

[12] Spiller HA, Bosse GM (2003). Prospective study of morbidity associated with snakebite envenomation. J Toxicol Clin Toxicol.

[13] Vickers AJ (1999). Comparison of an ordinal and a continuous outcome measure of muscle soreness. Int J Technol Assess Health Care.

[14] Hurst H, Bolton J (2004). Assessing the clinical significance of change scores recorded on subjective outcome measures. J Manipulative Physiol Ther.

[15] The Disabilities of the Arm, Shoulder and Hand Outcome Measure. [http://www.dash.iwh.on.ca/home].

[16] Beaton DE, Katz JN, Fossel AH, Wright JG, Tarasuk V, Bombardier C (2001). Measuring the whole or the parts? Validity, reliability, and responsiveness of the Disabilities of the Arm, Shoulder and Hand outcome measure in different regions of the upper extremity. J Hand Ther.

[17] Binkley JM, Stratford PW, Lott SA, Riddle DL (1999). The Lower Extremity Functional Scale (LEFS): scale development, measurement properties, and clinical application. North American Orthopaedic Rehabilitation Research Network. Phys Ther.

[18] Horn KK, Jennings S, Richardson G, Vliet DV, Hefford C, Abbott JH (2012). The patient-specific functional scale: psychometrics, clinimetrics, and application as a clinical outcome measure. J Orthop Sports Phys Ther.

[19] Stratford P (1995). Assessing disability and change on individual patients: a report of a patient specific measure. Physiother Can.

[20] Ware JEJ, Kosinski M, Bjorner JB, Turner-Bowker DM, Gandek B, Maruish ME (2007). User's manual for the SF-36v2^®^.

[21] Reilly MC, Zbrozek AS, Dukes EM (1993). The validity and reproducibility of a work productivity and activity impairment instrument. Pharmacoeconomics.

[22] WPAI Scoring. [http://www.reillyassociates.net/WPAI_Scoring.html].

[23] Figure of eight method of measuring ankle joint swelling. [http://www.physio-pedia.com/Figure_of_Eight_Method_of_Measuring_Ankle_Joint_Swelling].

[24] Leard JS, Breglio L, Fraga L, Ellrod N, Nadler L, Yasso M (2004). Reliability and concurrent validity of the figure-of-eight method of measuring hand size in patients with hand pathology. J Orthop Sports Phys Ther.

[25] Pellecchia GL (2003). Figure-of-eight method of measuring hand size: reliability and concurrent validity. J Hand Ther.

[26] Petersen EJ, Irish SM, Lyons CL, Miklaski SF, Bryan JM, Henderson NE (1999). Reliability of water volumetry and the figure of eight method on subjects with ankle joint swelling. J Orthop Sports Phys Ther.

[27] Jones LA (1989). The assessment of hand function: a critical review of techniques. J Hand Surg Am.

[28] Peters MJ, van Nes SI, Vanhoutte EK, Bakkers M, van Doorn PA, Merkies IS (2011). Revised normative values for grip strength with the Jamar dynamometer. J Peripher Nerv Syst.

[29] Bohannon RW (1997). Comfortable and maximum walking speed of adults aged 20–79 years: reference values and determinants. Age Ageing.

[30] Multiple Sclerosis Functional Composite (MSFC) Administration and Scoring Manual. [http://www.nationalmssociety.org/For-Professionals/Researchers/Resources-for-Researchers/Clinical-Study-Measures/Multiple-Sclerosis-Functional-Composite-(MSFC)].

[31] CFR - Code of Federal Regulations Title 21: 314.80 Postmarketing reporting of adverse drug experiences. [http://www.accessdata.fda.gov/scripts/cdrh/cfdocs/cfCFR/CFRSearch.cfm?fr=310.305].

[32] Harris PA, Taylor R, Thielke R, Payne J, Gonzalez N, Conde JG (2009). Research electronic data capture (REDCap)–a metadata-driven methodology and workflow process for providing translational research informatics support. J Biomed Inform.

[33] Hunsaker FG, Cioffi DA, Amadio PC, Wright JG, Caughlin B (2002). The American Academy of Orthopaedic Surgeons outcomes instruments: normative values from the general population. J Bone Joint Surg Am.

[34] Deshpande PR, Rajan S, Sudeepthi BL, Abdul Nazir CP (2011). Patient-reported outcomes: a new era in clinical research. Perspect Clin Res.

